# Steroid and Xenobiotic Receptor Signalling in Apoptosis and Autophagy of the Nervous System

**DOI:** 10.3390/ijms18112394

**Published:** 2017-11-11

**Authors:** Agnieszka Wnuk, Małgorzata Kajta

**Affiliations:** Institute of Pharmacology, Polish Academy of Sciences, Department of Experimental Neuroendocrinology, Smetna Street 12, 31-343 Krakow, Poland; wnuk@if-pan.krakow.pl

**Keywords:** apoptosis, autophagy, steroid receptors, xenobiotic receptors, nervous system, estrogen receptors

## Abstract

Apoptosis and autophagy are involved in neural development and in the response of the nervous system to a variety of insults. Apoptosis is responsible for cell elimination, whereas autophagy can eliminate the cells or keep them alive, even in conditions lacking trophic factors. Therefore, both processes may function synergistically or antagonistically. Steroid and xenobiotic receptors are regulators of apoptosis and autophagy; however, their actions in various pathologies are complex. In general, the estrogen (ER), progesterone (PR), and mineralocorticoid (MR) receptors mediate anti-apoptotic signalling, whereas the androgen (AR) and glucocorticoid (GR) receptors participate in pro-apoptotic pathways. ER-mediated neuroprotection is attributed to estrogen and selective ER modulators in apoptosis- and autophagy-related neurodegenerative diseases, such as Alzheimer’s and Parkinson’s diseases, stroke, multiple sclerosis, and retinopathies. PR activation appeared particularly effective in treating traumatic brain and spinal cord injuries and ischemic stroke. Except for in the retina, activated GR is engaged in neuronal cell death, whereas MR signalling appeared to be associated with neuroprotection. In addition to steroid receptors, the aryl hydrocarbon receptor (AHR) mediates the induction and propagation of apoptosis, whereas the peroxisome proliferator-activated receptors (PPARs) inhibit this programmed cell death. Most of the retinoid X receptor-related xenobiotic receptors stimulate apoptotic processes that accompany neural pathologies. Among the possible therapeutic strategies based on targeting apoptosis via steroid and xenobiotic receptors, the most promising are the selective modulators of the ER, AR, AHR, PPARγ agonists, flavonoids, and miRNAs. The prospective therapies to overcome neuronal cell death by targeting autophagy via steroid and xenobiotic receptors are much less recognized.

## 1. Introduction

It is generally accepted that mechanisms of neuronal demise involve apoptosis and autophagy. Apoptosis (“self-killing”) and autophagy (“self-eating”) are involved in neural development, as well as in the response of the nervous system to a variety of insults. Apoptosis is mainly responsible for cell elimination, whereas autophagy can either eliminate the cells or keep them alive, even in conditions lacking trophic factors. Therefore, both processes may act in the same direction or oppositely [1]. An excess of apoptosis or a defect in autophagy have been implicated in neurodegeneration. Autophagy is a basic cellular process that is crucial for postmitotic neurons, whereas apoptosis occurs at each stage of neural development and affects mitotically active and differentiated cells. Last year’s Nobel Laureate in Physiology or Medicine, Yoshinori Ohsumi, discovered and elucidated mechanisms underlying autophagy, a fundamental process for degrading and recycling cellular components, using baker’s yeast (The Nobel Prize in Medicine, 2016).

Apoptosis is considered to be the form of programmed cell death that is mediated via specific DNA fragmentation and apoptotic body formation. After initially being cut into pieces of 300–50,000 base pairs, DNA is cleaved by endonucleases (e.g., CAD - caspase-activated DNase) and Nuc-18 - endonuclease II) into pieces of 180–200 base pairs. In addition to apoptosis, this ladder-type DNA fragmentation is also found in some cells dying of necrosis [2]. Recent revelations suggest that apoptosis shares characteristics with necrosis, and these phenomena are interlinked in necroptosis. Apart from specific DNA fragmentation and apoptotic body formation, apoptosis is characterized by cell rounding, membrane blebbing, cytoskeletal collapse, cytoplasmic condensation and fragmentation, nuclear pyknosis, and individual cell death without inflammatory response to damage. Apoptosis has been documented to be involved in etiology of various types of neural degenerations, particularly these related to mitochondrial dysfunctions.

Autophagy is another form of programmed cell death that regulates lysosomal turnover of organelles and proteins via sequential events including double-membrane formation, elongation, vesicle maturation, and delivery of the targeted materials to the lysosome. This process is deleterious in acute neural disorders, such as stroke and hypoxic/ischemic injury [3]. However, autophagy appears protective in chronic neurodegenerative diseases such as Alzheimer’s disease (AD), Parkinson’s disease (PD), Huntington’s disease (HD), amyotrophic lateral sclerosis (ALS), and encephalopathy, where it is responsible for degrading not only damaged organelles, but also misfolded proteins [4]. Neural degeneration has been postulated to be associated with acceleration of apoptosis and impairment of autophagy, except for in cases of acute neural injury where both processes are stimulated.

The development of the nervous system is a highly complex process in which progenitor and stem cells differentiate into neurons, astrocytes and oligodendrocytes. During this process, not only does differentiation occur, but also the decisions of cell survival or cell death. The appropriate interplay between apoptosis and autophagy is believed to be essential for the normal development of the nervous system in mammals; therefore, neural development requires the degradation or subsistence of different organelles and proteins. Both major types of programmed cell death play roles in regulating neural cell numbers, tissue remodelling processes, and homeostasis. Apoptosis that occurs physiologically during the period of the growth spurt eliminates excessive neurons during the developmental process termed pruning [5]. Studies revealed an essential role of autophagy in the development and maturation of axons, dendrites and synapses [6].

Steroid and so-called xenobiotic receptors are involved in neural development; however, their actions as regulators of apoptosis and autophagy are complex. In general, estrogen, progesterone, and mineralocorticoid receptors mediate anti-apoptotic signalling, whereas androgen and glucocorticoid receptors participate in pro-apoptotic pathways. Estrogen receptors (ERs) play crucial roles in neurogenesis, astroglial proliferation and synaptogenesis. Neural progenitor cells (NPCs) express robust levels of aryl hydrocarbon receptor (AHR) that participate in NPC expansion and their differentiation into neurons. Retinoid X receptor (RXR) exerts its action by binding to gene sequences as either a homodimer or heterodimer and by regulating the transcription of specific genes. It forms RXR homodimers, such as RXRα, RXRβ, and RXRγ, and heterodimers, including the pregnane X receptor (PXR), constitutive androstane receptor (CAR), liver X receptor (LXR), and peroxisome proliferator-activated receptors (PPARs). All these receptors act as transcription factors, including the RXR-related xenobiotic receptors, which regulate neuronal differentiation both during development and adult neurogenesis. The RXR/nuclear receptor related 1 protein (NURR1) heterodimer has been postulated to be essential for the differentiation of the midbrain dopamine neurons [7].

Although steroid and xenobiotic receptors are essential for proper brain development, this review focused on the roles of steroid and xenobiotic receptors in apoptosis- and autophagy-related pathologies of the nervous system.

## 2. Molecular Mechanisms of Apoptosis and Autophagy

### 2.1. Mechanisms of Apoptosis

To identify potential drug targets, a major focus of neuroscience research is to examine the mechanisms involved in neuronal loss. Neurotrophins, such as nerve growth factors (NGFs), brain-derived neurotrophic factors (BDNFs), and neurotrophin-3 (NT-3), has been found to promote neuronal survival via RAS and PI-3K (3-phosphatydylinosytol kinase) pathways. Deficiency of neurotrophic factors inhibits PI-3K and promotes reactive oxygen species (ROS) production, which activates JNK (c-Jun N-terminal kinase)/SAPK (stress-activated protein kinase)-dependent apoptosis [2,8]. Genetic studies have demonstrated that the removal of specific *JNK* genes can reduce the neuronal death associated with cerebral ischemia [9]. A controversy has emerged regarding the question of whether limited neurotrophic factors are associated with the absence of inhibitors of cell death or if they are active signals of apoptosis. In general, apoptotic processes have been classified as extrinsic or intrinsic apoptotic pathways. The extrinsic pathway is induced by specific cell damage and is mediated through so-called “death receptors”, e.g., FAS, TNF-R1 (tumour necrosis factor receptor-1), TRAMP (death receptor 3/APO-3/LARD/wsl-1), TRAILR2 (death receptor 5/DR5), and DR6 (death receptor 6). The intrinsic pathway is initiated by non-specific cell damage that leads to the loss of the mitochondrial membrane potential, cytochrome c release from mitochondria and activation of the evolutionarily conserved cysteine-aspartic acid proteases-caspases. Mitochondrial membrane permeability to cytochrome c is primarily regulated by proteins from the BCL2 family, including anti-apoptotic (BCL2, BCLw, and BCLxL) and pro-apoptotic (BAX, BID, BAK, BAD, BOX, and BCLxS) proteins [10,11].

Apoptosis is usually a caspase-dependent process that depends on either the interaction of a death receptor with its ligand and subsequent activation of procaspase-8 or on the participation of mitochondria and the activation of procaspase-9. The main executioner protease of the apoptotic cascade is caspase-3, which activates CAD after cleavage of ICAD (inhibitor of caspase-activated DNase), thereby inducing apoptotic DNA fragmentation and apoptotic cell death [12,13]. In addition to their roles in apoptosis, executioner caspases (e.g., caspases-3, -6, and -7) have been recognized as important regulators of an array of cellular activities in the nervous system, including axonal pathfinding and branching, axonal degeneration, dendritic pruning, and microglial activation in the absence of death. Caspase activation has been postulated to be coordinated at multiple levels, which might underlie apoptotic and non-apoptotic roles of caspases in the nervous system. It has been shown that apoptosis may also be mediated by other cysteine-dependent proteases such as calpains, which are calcium-activated neutral proteases [14].

The intrinsic and extrinsic apoptotic pathways are regulated by p53, which is a cellular sensor for cell cycle and genomic stability. The most commonly inactivated tumour suppressor gene *p53* causes loss of p53 function, inhibits apoptosis, and promotes tumour progression and chemoresistance. Several proteins have been shown to interact with the p53 to regulate its functions. One of these regulatory proteins is glycogen synthase kinase 3 beta (GSK-3β), which binds to p53 and promotes p53-induced apoptosis [15]. GSK-3β is involved in modulating a variety of functions, including cell signalling, growth metabolism, DNA damage, hypoxia, and endoplasmic reticulum stress [16]. GSK-3β has been recognized as a primary kinase involved in tau hyperphosphorylation, and thus, it is responsible for neurodegenerative tauopathies, such as AD [17]. RNA interference silencing of GSK-3β has been found to inhibit the phosphorylation of tau protein, which may have a therapeutic effect on the pathological progression of AD [18]. Moreover, GSK-3β is involved in the accumulation of α-synuclein aggregates, oxidative stress and mitochondrial dysfunction, which make this kinase an attractive therapeutic target for neurodegenerative disorders, such as AD or PD [19].

**Figure 1:**
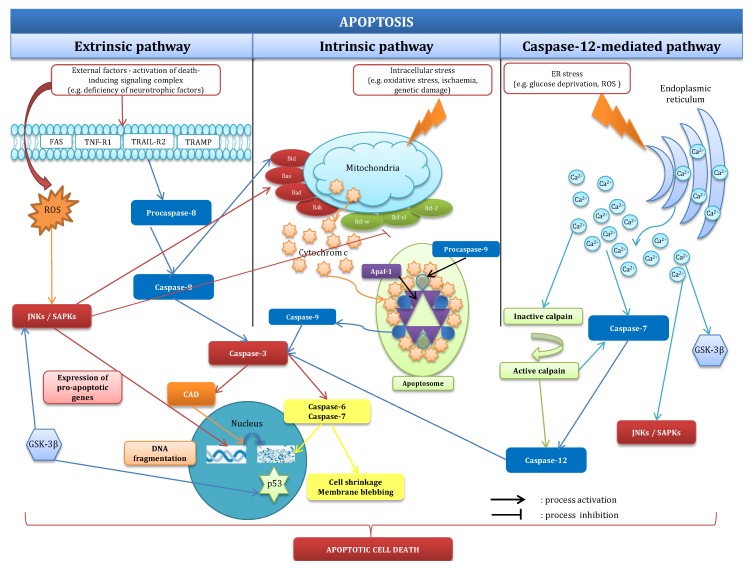
Mechanisms of apoptosis. Apoptosis has been classified as external, internal, and caspase-12-dependent processes. Additional details have been provided in part 2.1. CAD: caspase-activated DNase; GSK-3β: glycogen synthase kinase 3 beta; ROS: reactive oxygen species; JNK: c-Jun N-terminal kinase; SAPK: stress-activated protein kinase.

Apart from the intrinsic and extrinsic apoptotic pathways, there are also other pathways such as the caspase-12-mediated pathway, which is activated by calcium ions stored in the endoplasmic reticulum. Chronic or unresolved endoplasmic reticulum stress can induce neuronal apoptosis by activating JNK, GSK-3β, and the caspase-12 pathway [20]. The activated caspase cleaves procaspase-3 to induce classical apoptosis. Endoplasmic reticulum stress can be induced by a variety of physiological conditions, including perturbations in calcium homeostasis, glucose/energy deprivation, redox changes, ischemia, hyperhomocysteinemia, viral infections and mutations that impair protein folding. The endoplasmic reticulum stress response, also called the unfolded protein response, activates autophagy to remove aggregates of misfolded proteins that cannot be degraded. A previous study suggested that autophagy can provide neuroprotection by enhancing the clearance of these aggregates [21]. Recently, it has been shown that the tumour suppressor p53 can regulate cell death and autophagic activity, particularly mitophagy [22]. The p53 protein was found to be a cellular sensor of various stresses, including apoptotic stimuli. The induction of p53-dependent apoptosis leads to the activation of the intrinsic and extrinsic pathways to trigger cell death through transcription-dependent and transcription-independent mechanisms, among which is mitochondrial ROS production. In neurons, the extracellular signal-regulated kinases (ERKs) have been observed to mediate apoptosis; however, the majority of studies have demonstrated an anti-apoptotic role of ERK signalling. These processes have been visualized in Figure 1.

### 2.2. Mechanisms of Autophagy

Autophagy is a system of cellular degradation that ensures adequate digestion of cell debris and toxic material. The cytoplasmic debris is first enclosed in an autophagosome, which then fuses with the lysosome and forms an autophagolysosome for full digestion of the sequestered material. The formation of autophagosomes depends on several core Atg proteins, such as the following: ULK1 complex, Beclin1: Vps34/Atg14 L complex, and WIPIs, Atg12 and LC3 conjugation systems, and Atg9. In addition to the degrading function, autophagy promotes the recycling of cellular components, cellular renovation and homeostasis. Due to its important role, it is not surprising that autophagy dysregulation is found in many human diseases, such as cancer and neurodevelopmental and neurodegenerative diseases [23]. Recognition of the molecular mechanisms of autophagy processes was achieved through broad genetic research using mutagenesis in yeast cells, *Saccharomyces cerevisiae,* which allowed for the understanding of this complicated process in mammalian cells [24]. Autophagy can be divided into 3 stages: (1) formation of the autophagosome; (2) formation of the autophagolysosome; and (3) digestion the contents of follicles.

**Figure 2:**
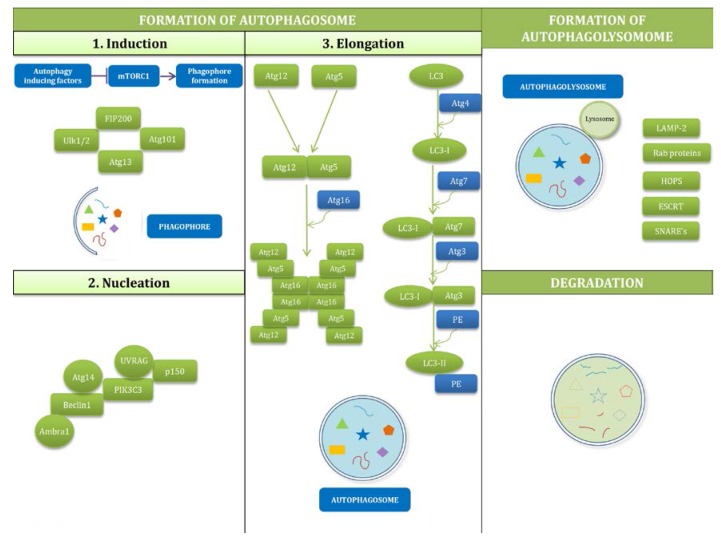
Mechanisms of autophagy. Autophagy can be divided into 3 stages: (1) formation of the autophagosome; (2) formation of the autophagolysosome; and (3) digestion the contents of follicles. Phagophore/autophagosome content: unneeded/misfolded proteins, carbohydrates, lipids, nucleic acids, whole organelles. More information has been provided in part 2.2.

Formation of the autophagosome involves induction, nucleation and elongation. Induction of autophagosome formation has its origins in co-assemblies of ULK1/2, FIP200, and Atg13 and Atg101 creating a so-called ULK1/2 complex. The initiation phase mainly depends on mammalian target of rapamycin (mTOR) kinase, which is a kind of sensor watching over the cell’s condition; however, it can also be induced by diverse input signals such as nutrients, growth factors, Ca^2+^, ATP, cAMP, hormones, and protein accumulation. Under nutrient-rich conditions, phosphoinositide 3-kinase (PIK3C1) activates mTORC1, which then phosphorylates ULK1 and Atg13 and inhibits autophagy. Under autophagy-inducing cases, such as starvation or hypoxia, mTORC1 is inactivated, and this results in activation of ULK1/2, which phosphorylates Atg13 and FIP200 and regulates proper localization of Atg9 and PIK3C3, both of which are essential in further steps of autophagy. For nucleation, at this stage, the most important factor is the complex of PIK3C3: Beclin1: p150-serine kinase. This complex is located at the phagophore and recruits other Atg proteins. PIK3C3 induces phosphatidyl-inositol-3-phosphate (PI3P) and the activity of this kinase is regulated by Beclin1, which interacts with multiple modulators. Elongation is a step of autophagosome formation that is based on two ubiquitin-like conjugation systems: Atg12-Atg5-Atg16 L (consisting of Atg5, Atg7, Atg10, Atg12, Atg16) and LC3-PE (composed of LC3, 3-phosphatidyl ethanolamine, Atg4, Atg7, Atg3). After formation of the autophagosome, it undergoes a process of maturation, i.e., a fusion with lysosome, thus forming the autophagolysosome [25]. It is a complicated and unclear action which requires several proteins such as LAMP-2, RAB proteins, SNAREs, ESCRT, and HOPS [26]. The fusion with lysosomes leads to the start of degradation of vesicle content by hydrolases and lipases. The final stage of autophagy is the efflux of autophagolysosome content into the cytoplasm. Scheme of these processes is presented in Figure 2.

### 2.3. Crosstalk between Apoptosis and Autophagy

**Figure 3:**
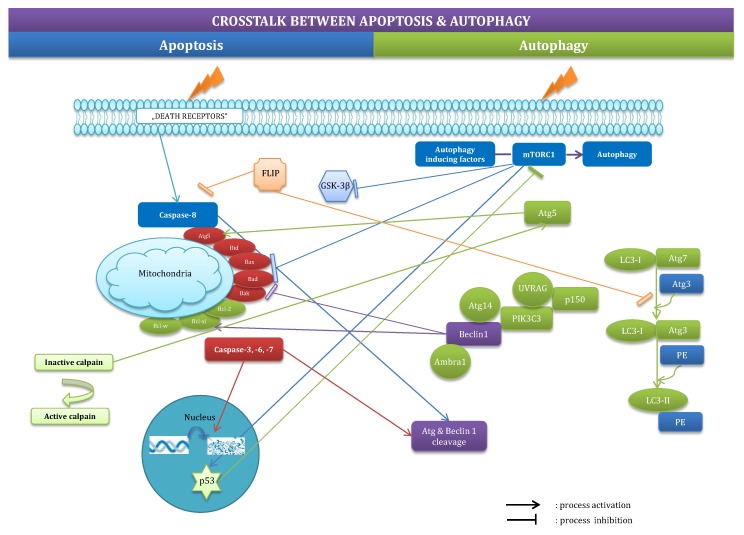
Crosstalk between apoptosis and autophagy. These processes interfere with themselves, mainly with regard to the BCL2 protein family, p53, and Atg5. The crosstalk has been described in detail in part 2.3.

The crosstalk between autophagy and apoptosis has only been partially uncovered. These processes interfere with themselves, mainly with regard to the BCL2 protein family, p53, and Atg5. Autophagy-related Beclin-1 inhibits or stimulates apoptosis, depending on the pro- or anti-apoptotic nature of the members of the BCL2 protein family with which it interacts [27]. Pro-apoptotic p53 initiates or inhibits autophagy depending on the cellular localization of the protein [22]. Atg5 participates in the formation of autophagosomes, translocates to the mitochondria and becomes a pro-apoptotic factor after catalytic incision by calpains [28]. In addition, mTOR, which is an inhibitor of autophagy, also acts on apoptosis-related p53, BAD and BCL2 [29]. Furthermore, caspases mediate cleavage of autophagy-related proteins, including Beclin-1, Atg4D, Atg8, and Atg5 [30]. The latest studies have demonstrated that the anti-apoptotic protein FLIP prevents elongation of autophagosomes by competing with LC3 for binding to Atg3 [31]. However, many questions regarding the interconnecting regulators of apoptosis and autophagy remain unanswered [1]. Crosstalk between apoptosis and autophagy has been outlined in Figure 3.

## 3. Interactions of Apoptosis and Autophagy with Steroid and Xenobiotic Signalling

### 3.1. Interactions with Estrogen Receptors

ER-dependent pathways stimulate neurotrophin expression, e.g., BDNF and glial cell-derived neurotrophic factor (GDNF), concomitantly with dendritic growth and spinogenesis [10]. In addition to beneficial effects of estrogens on cognitive dysfunction, estrogens were found to protect the functioning of GABAergic neurons [32]. A decline in ERα has been reported in the brains (hippocampus and frontal cortex) of individuals with schizophrenia and AD [33,34]. There was a greater accumulation of amyloid-beta (Aβ) in the brain of ERα knockout mice, and this accumulation greatly worsened memory when compared to control mice [35]. In rapidly autopsied human brain tissue, the frontal cortices of female AD patients exhibited significantly reduced mitochondrial ERβ compared to that in normal controls [36]. In the embryonic brain, ERβ appeared to be necessary for the development of calretinin-immunoreactive GABAergic interneurons and for neuronal migration in the cortex [37]. It became evident that a membrane-bound ER, GPR30, modulates synaptic plasticity in the hippocampus, and its deficiency in male mice results in insulin resistance, dyslipidemia, and a pro-inflammatory state [38].

Most of the biological effects of estrogens are mediated by the classical ERs: ERα and ERβ. The best recognized effects are the interactions between the ERs and the intrinsic mitochondrial apoptotic pathway [39]. There is a line of evidence that suggests that ERs directly interfere with BCL2-dependent apoptotic processes, namely, those observed in AD and PD. Neuroprotection against 1-methyl-4-phenyl-1,2,3,6-tetrahydropyridine- (MPTP-) or Aβ-induced toxicity was found to be mediated via ERα and ERβ, as well as by an increase in BCL2/BAD ratio [40,41]. The involvement of ERs in the inhibition of caspase- and GSK-3β-mediated neuronal cell death has also been shown, including in our studies [42,43,44]. Furthermore, ERs were found to suppress the extrinsic death receptor-mediated apoptotic pathway by decreasing the cell-surface expression of the Fas/Apo-1 receptor in neuroblasts [45]. Studies have shown that GPR30, also known as G-protein-coupled ER1 (GPER1), mediates non-genomic estradiol signalling in a variety of tissues, including the brain, with particularly high expression in the hypothalamus, hippocampus, cortex, and striatum [46,47]. Recently, GPR30 has been noticed to mediate the neuroprotective effects of estradiol in murine hippocampal and cortical cells [48,49]. It has also been found that GPR30 stabilized blood-brain barrier permeability after ischemic stroke, improved cognitive function that was impaired by traumatic brain injury, and inhibited PD-related neuroinflammation [50,51,52]. 

Our data demonstrated a key involvement of GPR30 and/or ERβ in the neuroprotective and anti-apoptotic actions of the phytoestrogens daidzein and genistein [53,54]. Similarly, ERβ signalling has been linked to flavonoid troxerutin-induced mitigation of apoptosis in a 6-hydroxydopamine-induced lesion rat model for PD [55]. In addition, we provided evidence for a crucial role of ERα in the neuroprotective function of raloxifene during hypoxia [56]. According to our in vitro data, the protective action of the selective estrogen receptor modulator (SERM) is mediated by ERα, but not by ERβ or GPR30 and involves the inhibition of apoptosis. This is in line with results from Guo et al., 2016 that showed the enhancement of the ERα-mediated transactivation of the BCL2 gene upon ischemic insult and that provided in vitro evidence that metastasis-associated protein 1 (MTA1) enhances the binding of ERα with the BCL2 promoter via recruitment of HDAC2, together with other unidentified coregulators [57]. In male Wistar rats subjected to transient right middle cerebral artery occlusion (tMCAO), Jover-Mengual et al., 2017 detected the increased expression of ERα or ERα and ERβ in response to estradiol and the SERM bazedoxifene, respectively [58]. In the most recent study, Guo et al., 2017 linked estradiol neuroprotection against ischemic brain injury and apoptosis to the SIRT1-dependent adenosine monophosphate (AMP)-activated kinase (AMPK) pathway [59].

The protective action of notoginsenoside R1 against cerebral hypoxic-ischemic injury was found to be the result of the estrogen receptor-dependent inhibition of endoplasmic reticulum stress pathways, involving a caspase-12-mediated apoptosis, and the activation of Akt/Nrf2 pathways [60,61]. The ginsenoside Rg1 protection against Aβ peptide-induced neuronal apoptosis was shown to involve ERα, as well as the up-regulation of ERK1/2 phosphorylation and the reduction of NF-κB nuclear translocation [62]. Additionally, the pharmacological administration of the isoflavone daidzein was shown to stimulate cell proliferation and inhibit high fat diet-induced apoptosis and gliosis in the rat hippocampus [63]. Furthermore, it became evident that the impairments of ERα and/or GPR30 participate in mechanisms of apoptotic actions of DDT and a chemical UV-filter benzophenone-3 (BP-3) [43,64]. Autosomal dominant optic atrophy, a progressive blinding disease featured by retinal ganglion cell degeneration, has recently been linked to the inhibition of the estrogen receptor expression, which promoted apoptosis in mouse females carrying the human recurrent OPA1 mutation [65].

Estrogen receptors are important regulators of neuronal apoptosis; however, little is known about their impact on autophagy. Knockdown of ERα or antagonizing GPR30 has been found to induce autophagy and apoptosis in cancer cells [66,67]. The phytoestrogen gypenoside XVII (GP-17) attenuated the Aβ25-35-induced parallel autophagic and apoptotic death of NGF-differentiated PC12 cells through the ER-dependent activation of the Nrf2/ARE pathways [68]. In SH-SY5Y cells, ERs mediated Aβ degradation via the up-regulation of neprilysin and promoted autophagy as a protective mechanism against chronic minimal peroxide treatment [69]. Moreover, ER agonists activated PI3K/Akt/mTOR signalling in oligodendrocytes to promote remyelination in a mouse model of multiple sclerosis (MS) [70,71], and the up-regulation of the membrane ERα was associated with functional signals that were compatible with autophagic cytoprotection of neuronal cell line SH-SY5Y.

### 3.2. Interactions with Androgen Receptors

Dehydroepiandrosterone (DHEA), also known as androstenolone, binds with high affinity to androgen receptors; however, it may also activate the ER, PXR, CAR, and PPARα. There is a body of evidence showing that DHEA activates Akt in neural precursors, in association with inhibition of apoptosis [72]. However, its sulfated derivative, DHEA, has the opposite effect of DHEA during neurogenesis. An anabolic-androgen 17β-trenbolone was found to induce apoptosis in primary hippocampal neurons that was accompanied by the up-regulation of β-amyloid peptide 42 (Aβ42) and the activation of caspase-3 [73]. Androgen-induced neurotoxicity has been detected in the dopaminergic cell line N27 [74]. Following treatment with testosterone or dihydrotestosterone, the cells exhibited mitochondrial dysfunction and died due to apoptosis. Androgens appeared to have both neuroprotective and neurotoxic effects depending on age and sex, as evidenced by examining the effect of androgens on cell survival after an excitatory stimulus in the developing hippocampus in both males and females [75]. Pike et al., 2008 discussed the involvement of androgen cell signalling pathways in neuroprotection, showing, as an example, the age-related testosterone loss in men with increased risk for AD [76].

### 3.3. Interactions with Progesterone Receptors

Many studies have shown that progesterone promotes the viability of neurons in the brain and spinal cord. Progesterone appeared particularly effective in treating traumatic brain and spinal cord injuries, as well as ischemic stroke [77]. Moreover, progesterone has been shown to attenuate Aβ (25–35)-induced neuronal toxicity via JNK inactivation and the inhibition of mitochondrial apoptotic pathway by progesterone receptor membrane component 1 [78]. Studies using yeast as a model system extended the protective actions of progesterone to the reduction of cytosolic calcium and the reduction in ROS production and ATP levels; however, these effects did not depend on the yeast orthologue of the progesterone receptor, Dap1 [79]. Progesterone receptor membrane component 1 (PGRMC1) is a key regulator of apoptosis, and its up-regulation was found in retinal degeneration 10 (rd10) mice, which are a model system for autosomal recessive retinitis pigmentosa [80].

### 3.4. Interactions with Glucocorticoid and Mineralocorticoid Receptors

Previous studies have shown that dexamethasone-induced apoptosis of primary hippocampal neurons involved GR and was counteracted by an MR agonist aldosterone [81]. In general, GR activation has been implicated in the induction of an endangered neural phenotype, whereas MR expression appeared to be associated with a neuroprotective phenotype [82]. When used therapeutically to treat respiratory dysfunction associated with premature birth, the endogenous rodent glucocorticoid corticosterone has been shown to activate GR and cause progenitor cell apoptosis, as well as neurodevelopmental deficits, particularly in the cerebellum [83]. In contrast, MR overexpression inhibited apoptosis and promoted survival of embryonic stem cell-derived neurons [84]. Unlike in other brain regions, glucocorticoids play a critical role in retinal photoreceptor survival, whereas mifepristone, which has the capacity to block glucocorticoid receptors, promotes photoreceptor death [85]. Furthermore, an MR agonist aldosterone appeared to be a critical mediator of retinal ganglion cell loss that was independent of elevated intraocular pressure [86].

### 3.5. Interactions with Aryl Hydrocarbon Receptor

There are data, including ours, that have revealed that the aryl hydrocarbon receptor (AHR) regulates apoptosis in the mammalian brain [42,43,87,88,89,90]. Previously, we demonstrated that the selective AHR agonist, β-naphthoflavone, induced caspase-3-dependent apoptosis in primary cultures of mouse neurons. This effect was accompanied by the increased expression of AHR that co-localized with ERβ, thus supporting the direct interaction between AHR-mediated apoptosis and ERs signalling [42]. We also showed the involvement of AHR in apoptotic and neurotoxic actions of the pesticide dichlorodiphenyltrichloroethane (DDT) and the antimicrobial agent triclosan [43,90]. In addition, we detected the enhanced mRNA and protein expression levels of AHR in one-month-old mice that were prenatally exposed to DDT [91]. Since enhanced AHR expression was accompanied by DNA hypomethylation, both global DNA and the DNA of the specific *AHR* gene, we hypothesized that AHR signalling leads to apoptosis that underlies the fetal basis of the adult onset of disease. In our study, we showed that by targeting AHR/AHR nuclear translocator (ARNT) signalling, 3,3’-diindolylmethane (DIM) inhibited caspase-3-dependent apoptosis and rescued neurons from hypoxia [89]. This effect was followed by a decrease in the expression levels of AHR, AHR-regulated cytochrome P450 1A1 (CYP1A1), and ARNT.

### 3.6. Interactions with RXR-Related Xenobiotic Receptors

Recently, we showed that CAR, PXR, and RXRs were involved in nonylphenol-initiated apoptosis in mouse hippocampal neurons in primary cultures [44,92]. We also found that RXRs participated in the propagation of DDE- and BP-3-induced neuronal apoptosis [93,94], whereas PPARγ was impaired in response to apoptotic actions of BP-3, dibutyl-phthalate, and tetrabromobisphenol A [64,95,96]. Interestingly, the activation of CAR by the CITCO agonist increased ABC-transporter expression (Abcb1 and Abcg2) in blood-brain barrier and inhibited growth and expansion of brain tumour stem cells via inducing cell cycle arrest and apoptosis [97,98]. Similarly, a PPARα-selective activator 4-chloro-6-(2,3-xylidino)2-pyrimidinylthioacetic acid (Wy-14,643) enhanced cell death in cultured cerebellar granule cells [99]. On the other hand, RXR activation was essential for docosahexaenoic acid to protect retina photoreceptors against oxidative stress (paraquat, H_2_O_2_)-induced apoptosis [100,101], and the addition of 9-*cis*-retinoic acid prevented dimerization of RXR and a nerve growth factor-induced clone B (NGFI-B) that rescued cerebellar granule neurons from calcium-induced apoptosis [102]. An RXR agonist bexarotene was found to up-regulate the lncRNA Neat1 and to inhibit apoptosis in mice after traumatic brain injury [103]. Moreover, activated LXR inhibited a 7-ketocholesterol-induced apoptosis in human neuroblastoma SH-SY5Y cells [104]. Mutation of NR4A2 (also called NURR1), a RXR-partner for heterodimerization, is involved in familiar form of PD. In patients with PD, NR4A2 expression is downregulated, whereas in patients with AD reduced NR4A1 level is observed [105]. Additionally, NR4A has been identified as a key regulator of catecholamine production by macrophages and the mediator of CREB-induced neuronal survival [106,107].

## 4. The Roles of Apoptosis and Autophagy in Pathologies of the Nervous System

### 4.1. Apoptosis in Pathologies of the Nervous System

Apoptosis is involved in the pathogenesis of neurodegenerative diseases, such as stroke, HD, AD, and PD. The accumulation of misfolded Aβ and α-synuclein, two major toxic proteins in AD and PD, leads to neuronal apoptosis. There is increasing evidence that caspase-6 is highly involved in axon degeneration in HD and AD. Active caspase-6 has been found in the early stages of AD [108], and cleavage at the caspase-6-cleavage site in mutated huntingtin protein is a prerequisite for the development of the features of HD [109]. The PTEN-induced kinase 1 (PINK1) that is linked to the autosomal recessive familial form of PD has been found to protect cells from mitochondrial dysfunction and is a key player in many signalling pathways in response to oxidative stress, including apoptosis [110]. A previous study supports an important role of ceramides in neuronal apoptosis, particularly their role in the stabilization of β-secretase, amyloidogenic processing of Aβ precursor protein (APP), and generation of Aβ, which is the major component of the senile plaques [111].

Currently, there has been considerable effort directed towards GSK-3β as a potential target for the treatment of many diseases, including Type-II diabetes and neurodegenerative diseases [112]. Activated GSK-3β has been reported to induce apoptosis in neurons [113,114] and to regulate tau phosphorylation and Aβ peptide production. Extracellular deposits of Aβ and intracellular deposits of hyperphosphorylated tau protein are major histopathological hallmarks of AD. Because GSK-3β phosphorylates tau proteins, it is thought that disruption of GSK-3β signalling may contribute to the onset of the disease. Targeting this enzyme has been found to inhibit the symptoms of PD, such as enhanced expression of α-synuclein and the loss of dopaminergic neurons in the substantia nigra pars compacta [115]. We previously showed that the neuroprotective action of genistein is mediated by the inhibition of GSK-3β signalling [53]. Our recent study demonstrated that the pesticide DDT-induced apoptosis of mouse neurons is a caspase-9-, caspase-3-, and GSK-3β-dependent process [43]. By activating GSK-3β and JNK, the endoplasmic reticulum stress can induce caspase-12-dependent apoptosis, which has been implicated in a broad range of human diseases, including neurodegenerative diseases, cancer, diabetes, and vascular disorders.

Preclinical data showed that, apart from the inhibition of excitatory activity of neurons via modulation of two major neurotransmitter receptor groups, i.e., *N*-methyl-d-aspartate (NMDA) receptors, and γ-aminobutyric acid (GABA) receptors, anasthetics caused apoptosis and neurodegeneration in the developing brains of neonates [116]. These effects could lead to learning and memory deficits, as well as abnormalities in social memory and social activity. Similarly, glucocorticoid therapy, which was invented to accelerate lung maturation and reduce inflammation in newborns, was also found to induce apoptosis in the cerebellar external granule layer. The therapy caused a disruption of cerebellar development that was followed by neuromotor and cognitive deficits [117].

### 4.2. Autophagy in Pathologies of the Nervous System

Dysregulation of autophagy is involved in the etiology of neurodevelopmental diseases, such as autism and fragile X syndrome [23]. Global knockout of *Beclin 1*, *Ambra*, *Atg5*, or *Atg7* is lethal and causes death within a few days after birth [118]. The impairment of autophagy has been postulated to be involved in the onset of neural degeneration in PD, AD, and HD, as well as the degeneration accompanying ALS and MS [4]. An age-related decrease in the expression of the indispensable autophagy protein, Beclin1, has been suggested to have consequences for mHtt accumulation in HD [119]. Selective inhibition of autophagy with 3-MA and chloroquine has been shown to cause a delay in tau clearance, and *Atg7* knockout mice were shown to exhibit excessive amount of phosphorylated tau that is typical for AD [120]. Moreover, the failure of autophagy or mutations in *Parkin* and *PINK1* have been found to result in abnormal autophagic degradation of α-synuclein aggregates [121].

The dysfunction of mitochondria that intensively influences the autophagy pathway is one of the important factors in the pathogenesis of MS [122]. Administration of rapamycin, an inhibitor of mTOR, ameliorates relapsing-remitting experimental autoimmune encephalomyelitis (EAE). However, ALS-associated autophagy remains controversial, and it is still not known whether activating autophagy is beneficial or harmful for motor neuron degradation. Studies reported that toxic accumulation of mutant superoxide dismutase (SOD1) protein contributes to autophagy by its retardation [123]. In addition, it has been found that autophagy may be pro- or anti-inflammatory. The interplay between the microglial inflammatory response and microglial autophagy is inherent to acute central nervous system (CNS) injury as well as the recovery stage of chronic CNS injury [124].

Studies have suggested that autophagy can provide neuroprotection by enhancing the clearance of misfolded protein aggregates [21]. There are, however, data that indicated that autophagy acts in parallel with neurodegenerative processes initiated by kainic acid and hypoxia [125,126]. Recent advances in neurodegenerative models associated with the formation of protein aggregates, such as PD, HD, and AD, target autophagy by treatment with the mTOR inhibitor rapamycin or analogues to force the degradation of potentially toxic aggregates. Surprisingly, the inhibition of mTOR has been found to cause neural degeneration, whereas excessive activity of the kinase was found to impair neural development and lead to neuroectodermal dysplasia [127]. Recent studies have demonstrated that cellular autophagy markers are up-regulated in humans upon treatment with antidepressants [128]. One may assume that autophagy might be a double-edged sword in major depressive disorder (MDD), which suggests why some MDD patients remain resistant to certain antidepressant medications.

## 5. Perspectives Related to Targeting Apoptosis and Autophagy via Steroid and Xenobiotic Receptor Signalling

### 5.1. Targeting Apoptosis

#### 5.1.1. Via Estrogen Receptors

There is a wealth of information indicating that estrogens exert actions involved in neuroprotection. They protect neurons against different insults, such as anoxia, oxidative stress, glutamic acid, hydrogen peroxide, iron, and the Aβ peptide. However, the application of estrogens as neuroprotectants in humans presents numerous limitations mainly due to the endocrine actions of the molecules on peripheral tissues and the increased frequency of hormone-dependent tumours as well as cerebrovascular disease and stroke risk. In addition, the highly oxidative cellular environment present during neurodegeneration stimulates the hydroxylation of estradiol to the catechol-estrogen metabolites, which can undergo reactive oxygen species-producing redox cycling, setting up a self-generating toxic cascade [129].

The conflict between basic scientific evidence for estrogen neuroprotection and the lack of effectiveness in clinical trials is only now being resolved. From birth to menopause, the ovaries produce high circulating levels of estradiol, which correlates with a low incidence of neurodegenerative disease. Once the menopausal transition occurs, the risk for neurodegenerative diseases, including ischemic stroke and AD, increases. Although the Women’s Health Initiative Memory Study (WHIMS) pointed to some cognitive adverse effects of postmenopausal hormone therapy, these results were not relevant to peri- and early menopause, since WHIMS recruited women above the age of 65 years [130]. Emerging evidence from basic science and clinical studies suggests that there is a “critical period” for the beneficial effect of estradiol on the brain. The critical window hypothesis suggests that initiating hormone therapy at a younger age in closer temporal proximity to menopause may reduce the risk of AD [131]. This is in accordance with a transcriptome meta-analysis that revealed a central role for sex steroids in the degeneration of hippocampal neurons in AD [132]. Currently, there is a need for a new, safe, and effective ER-dependent therapy.

The possibility of using SERMs to induce estrogen-like protective actions in the brain has emerged as an alternative to estrogen treatment. SERMs have been found to trigger neuroprotective mechanisms that reduce neural damage in different experimental models of neural trauma, brain inflammation, and neural degeneration. Most studies have focused on tamoxifen and raloxifene, which have been used in human clinics for years. Their neuroprotective actions have been assessed in different experimental models of neural dysfunction. These include models of traumatic injury to the central nervous system and peripheral nerves, stroke, MS, and PD and AD [133]. The best documented neuroprotective actions are antioxidant effects and the prevention of excitotoxicity, which is a common cause of neuronal death in neurodegeneration [134]. Some key molecules have been identified, such as mitogen-activated protein kinases (MAPK), PI3K/Akt, cAMP response element binding protein (CREB), and NF-κB [135,136,137], but the precise molecular targets and mechanisms involved in the neuroprotective actions of SERMs need to be determined. Our recent study demonstrated protective, including anti-apoptotic, effects of raloxifene on brain neurons subjected to hypoxia [56]. We showed that raloxifene-induced neuroprotection almost exclusively depended on ERα but not ERβ and GPR30.

Currently, a major focus of neuroscience research is to examine the mechanisms involved in neuronal loss that will be necessary to identify potential drug targets. These include microRNA, such as miR-7-1, which enhanced the neuroprotective effects of estrogen receptor agonists, i.e., 1,3,5-*tris*(4-hydroxyphenyl)-4-propyl-1*H*-pyrazole (PPT), Way 200070, and estrogen, in preventing apoptosis in A23187 calcium ionophore-exposed VSC4.1 motoneurons [138]. Targeting ERβ, with a combination of natural estrogen-like compounds that bind the receptor with high selectivity, has been proposed as a therapeutic strategy for Leber’s hereditary optic neuropathy [139]. ERβ ligand AC186 is a new candidate for neuroprotection in MS [140]. Following HIV-1 Tat (1–86) exposure, soy isoflavones induced anti-apoptotic actions in neurons by targeting ERs [141]. Purple sweet potato colour significantly suppressed endoplasmic reticulum stress-induced apoptosis and promoted ERα-mediated mitochondrial biogenesis in mice challenged with domoic acid [142]. An inverted correlation between the levels of ERα and parkin in the striatum of adult mice suggests a possible role of the receptor in preventing the parkin-related PD in humans [143]. The discovery of a binding site of ERβ in the *Tnfaip1* (Tumour necrosis factor-induced protein 1) promoter region points to a novel regulatory site that could be targeted by estrogen or other selective ligands to protect the brain against AD and apoptosis [144]. A new strategy for neuroprotection against ischemic insults could involve targeting a novel 36-kDa variant of ERα, i.e., ERα36, which is associated with the phosphorylation of Akt in the cells exposed to glucose deprivation [145]. Targeting ERβ by *Cicer microphyllum* seed extract could rescue neurons from global hypoxia [146].

Up-regulation of neuroglobin upon 17β-estradiol (E2) stimulation has recently been linked to neuroprotection. After relocalization to the mitochondria, neuroglobin associates with cytochrome c, which reduces cytochrome c release into the cytosol, and subsequently inhibits caspase-3 activation and apoptotic cell death. Neuroprotection could also be directed to seladin-1 (selective AD indicator-1), which is down-regulated in the brain regions affected by AD and confers protection against β-amyloid-induced toxicity via the inhibition of caspase-3 activity. A seladin-1 gene has been found to be identical to the gene encoding the enzyme, 3-β-hydroxysterol Δ^24^-reductase, which is up-regulated by estrogen and involved in the cholesterol biosynthetic pathway [147].

#### 5.1.2. Via Androgen, Progesterone, and Corticoid Receptors

It recently became evident that the selective androgen receptor modulator (SARM), RAD140, has the capacity to protect cultured neurons and brain tissue against kainate-induced apoptosis [148]. This action depended on MAPK signalling, including ERK phosphorylation, and showed the relevance of androgen signalling to neural health and resilience to neurodegeneration. Progesterone was found to play a neuroprotective role in various models of neurodegeneration, including AD, through the inhibition of the mitochondrial apoptotic pathway, and by blocking Aβ-induced JNK activation [78]. A synthetic form of the female hormone progesterone, Norgestrel, which acts via progesterone receptor membrane component 1, has been proposed as therapeutic for the treatment of retinitis pigmentosa [80]. In patients with AD, increased expression of the translocator protein (TSPO; the former peripheral benzodiazepine receptor or PBR) shows another possibility for neuroprotection that could be executed via TSPO-mediated stimulation of steroid synthesis; as such, its neuroprotective and neuroregenerative properties resulted in the inhibition of apoptotic cell death. A novel TSPO (18 kDa) ligand, ZBD-2, which is involved in the synthesis of endogenous neurosteroids (e.g., pregnenolone, DHEA, and progesterone), effectively prevented NMDA-induced excitotoxicity and apoptosis and protected mouse brains against focal cerebral ischemia [149]. Recent studies provided evidence that corticosterone-induced injury in rat adrenal pheochromocytoma PC12 cells can be attenuated by HBOB, an HDAC6 inhibitor, by inhibiting mitochondrial GR translocation and the intrinsic apoptosis pathway [150]. Furthermore, agonizing the MR with fludrocortisone promoted cell survival and proliferation of adult hippocampal progenitors, but inhibited apoptotic signalling, including GSK-3β [151].

#### 5.1.3. Via AHR

Flavonoids have been shown to inhibit the development of AD-like pathology, and red wine consumption appeared to reduce age-related macular degeneration, stroke, and cognitive deficits. In addition to well documented free radical scavenging and anti-inflammatory properties, resveratrol, which is one of the key ingredients responsible for the neuropreventive action of red wine, has been shown to act as AHR antagonist and an inhibitor of apoptosis. The recent study of our group has shown the strong neuroprotective capacity of a selective aryl hydrocarbon receptor modulator (SAHRM) DIM against the hypoxia-induced damage in mouse hippocampal cells in primary cultures [89]. DIM-evoked neuroprotection was mediated by the impairment of AHR/ARNT signalling, as evidenced by the use of specific siRNAs, as well as quantitative (qPCR, ELISA) and qualitative (western blot analysis and confocal microscopy) assessments. Neuroprotective properties of DIM have also been shown in the cellular and animal models of PD [152]. Exciting prospects to overcome the cellular mechanisms that lead to neuronal injury involving apoptosis and autophagy have recently been related to Wnt (wingless-type) signalling, which is known to interact with AHR during neural development [153].

#### 5.1.4. Via Xenobiotic Receptors

In addition to the steroid receptor- and AHR-mediated signalling, xenobiotic receptors have become attractive mediators that can cause neuroprotection through interaction with the apoptotic pathways. PPARγ agonists (derivatives of thiazolidinediones, e.g., troglitazone, rosiglitazone and pioglitazone) have been associated with neuroprotection in different neurological pathologies, including AD and PD, cerebral ischemia, MDD and stroke [154,155,156,157]. However, the mechanisms involved in PPARγ effects in the nervous system are still unknown. PPARs and many other nuclear receptors form heterodimers with RXRs, and these heterodimers regulate the transcription of various genes. The activation of RXR/PPARγ by bexarotene was found to have neuroprotective potential in mice subjected to focal cerebral ischemia [158]. UAB30, a novel RXR agonist that induces apoptosis in human neuroblastoma, has also been shown to have a potential therapeutic role [159]. Recently, treatments with RXR agonists (bexarotene and fluorobexarotene) were found to promote Aβ degradation and rapidly reversed Aβ-induced behavioural deficits in AD [160,161,162]. Bexarotene was also found to protect dopaminergic neurons in animal models of PD [163].

### 5.2. Targeting Autophagy

In comparison to apoptosis, there is very little known about the interactions between the steroid and xenobiotic receptor signalling pathways and the process of autophagy. Therefore, targeting autophagy via steroid and xenobiotic receptors needs to be further elucidated. Estradiol has been shown to inhibit autophagy in the hippocampus CA1 region and to alleviate neurological deficits following cerebral ischemia [164]. The estrogen receptor and the estrogen-related receptor antagonists, tamoxifen, and 4-hydroxytamoxifen, were shown to induce cytotoxic autophagy in glioblastoma. However, the effect of 4-hydroxytamoxifen in malignant peripheral nerve sheath tumour cells did not depend on ER signalling, but on the degradation of the pro-survival protein Kirsten rat sarcoma viral oncogene homologue [165]. According to Felzen et al.2015, ERα-expressing neuroblastoma cells have a higher autophagic activity than cells expressing ERβ or lacking ER expression [166]. This new non-canonical autophagy is mediated by ERα, but it is estrogen response element (ERE)-independent and involves the function of the co-chaperone BCL2-associated athanogene 3 (BAG3). In studies using the SH-SY5Y cell line, mERα has been postulated to promote the maturation of autophagosomes into functional autolysosomes by regulating ERK [167].

## 6. Perspectives Related to Targeting Specific miRNAs which Interact with Steroid and Xenobiotic Receptor Signalling

MicroRNAs (miRNAs) are small non-coding RNA molecules that are almost exclusively negative regulators of gene expression in the nervous system. It has been shown that miR-218-affected tau phosphorylation is oppositely-regulated by classical estrogen receptors. ERα was found to increase the expression of miR-218 that was followed by diminished protein expression of tyrosine phosphatase alpha (PTPα), as well as by activation of GSK-3β and inactivation of protein phosphatase 2A, the major tau enzymes involved in AD pathology. In contrast, ERβ reduced miR-218 levels that resulted in inhibition of tau phosphorylation [168]. Recently, Micheli et al., 2016 demonstrated that 17β-estradiol-evoked neuroprotection from the Aβ-induced neurotoxicity was mediated by an increase in miR-125b expression and subsequent decrease in mRNA and protein expression of pro-apoptotic factors BAK1 and p53 [169]. Furthermore, an involvement of estrogen receptors in regulation of specific miRNAs in response to cerebral ischemia has been demonstrated. 17β-estradiol alone or in combination with progesterone was found to upregulate miR-375 expression and its target BCL2 in rat model of cerebral ischemia [170]. MiR-375 was also positively regulated by ERα in response to a phytoestrogen-calycosin that caused protection against cerebral ischemia [171]. Recently, the roles of estrogen and glucocorticoid receptors in regulation of cerebral miRNAs have been supported by contribution of miR-23a and miR-210 in response to cerebral ischemia [172,173]. Glucocorticoid-mediated attenuation of BDNF-dependent neuronal function has been linked to reduced expression of miR-132 [174]. The most relevant reports on AHR- and CAR-regulated miRNA showed the inversely co-related expression of AIP (AHR-interacting protein) and miR-107 in pituitary adenomas, as well as CAR and miR-137 in neuroblastoma cells [175,176].

## Abbreviations

AD	Alzheimer’s disease
AHR	aryl hydrocarbon receptor
AIP	AHR-interacting protein
ALS	amyotrophic lateral sclerosis
AMP	adenosine monophosphate
AMPK	adenosine monophosphate activated kinase
APP	Aβ precursor protein
AR	androgen receptor
ARNT	AHR nuclear translocator
Aβ	amyloid-beta
Aβ42	β-amyloid peptide 42
BAG3	BCL2-associated athanogene 3
BDNF	brain-derived neurotrophic factors
BP-3	benzophenone-3
CAD	caspase-activated DNase
CAR	constitutive androstane receptor
CNS	central nervous system
CREB	cAMP response element binding protein
CYP1A1	cytochrome P450 1A1
DDT	dichlorodiphenyltrichloroethane
DHEA	dehydroepiandrosterone
DIM	3,3′-diindolylmethane
DR6	death receptor 6
E2	17β-estradiol
EAE	experimental autoimmune encephalomyelitis
ER	estrogen receptor
ERE	estrogen response element
ERK	extracellular signal-regulated kinase
GABA	g-Aminobutyric acid
GDNF	glial cell-derived neurotrophic factor
GP-17	gypenoside XVII
GPER1	G-protein-coupled ER1, membrane-bound estrogen receptor
GPR30	membrane-bound estrogen receptor, also known as GPER1
GR	glucocorticoid receptor
GSK-3β	glycogen synthase kinase 3 beta
HD	Huntington’s disease
HDAC	histone deacetylases
ICAD	inhibitor of caspase-activated DNase
JNK	c-Jun N-terminal kinase
LXR	liver X receptor
MAPK	mitogen-activated protein kinases
MDD	major depressive disorder
MiRNA	microRNA
MPTP	1-Methyl-4-phenyl-1,2,3,6-tetrahydropyridine
MR	mineralocorticoid receptor
MS	multiple sclerosis
MTA1	metastasis-associated protein 1
mTOR	mammalian target of rapamycin kinase
NGF	nerve growth factors
NGFI-B	nerve growth factor-induced clone B
NMDA	*N*-methyl-d-aspartate
NPC	neural progenitor cells
NT-3	neurotrophin-3
NURR1	nuclear receptor related 1 protein
PD	Parkinson’s disease
PGRMC1	progesterone receptor membrane component 1
PI-3K	3-Phosphatydylinosytol kinase
PI3P	phosphatidyl-inositol-3-phosphate
PIK3C1	phosphoinositide 3-kinase
PINK1	PTEN-induced kinase 1
PTPα	tyrosine phosphatase alpha
PPAR	peroxisome proliferator-activated receptor
PR	progesterone receptor
PXR	pregnane X receptor
rd10	retinal degeneration 10
ROS	reactive oxygen species
RXR	retinoid X receptor
SAHRM	selective aryl hydrocarbon receptor modulator
SAPK	stress-activated protein kinase
SARM	selective androgen receptor modulator
seladin-1	selective AD indicator-1
SERM	selective estrogen receptor modulator
SOD1	superoxide dismutase
tMCAO	transient right middle cerebral artery occlusion
Tnfaip1	tumour necrosis factor-induced protein 1
TNF-R1	tumour necrosis factor receptor-1
TRAILR2	death receptor 5/DR5
TRAMP	death receptor 3/APO-3/LARD/wsl-1
TSPO	translocator protein
WHIMS	women’s health initiative memory study
Wnt	wingless-type
